# A Novel SRP Recognition Sequence in the Homeostatic Control Region of Heat Shock Transcription Factor σ^32^

**DOI:** 10.1038/srep24147

**Published:** 2016-04-07

**Authors:** Ryoji Miyazaki, Takashi Yura, Takehiro Suzuki, Naoshi Dohmae, Hiroyuki Mori, Yoshinori Akiyama

**Affiliations:** 1 Institute for Virus Research, Kyoto University, Kyoto 606-8507, Japan; 2 Faculty of Life Sciences, Kyoto Sangyo University, Kyoto 603-8555, Japan; 3 Center for Sustainable Resource Science, RIKEN, Saitama 351-0198, Japan

## Abstract

Heat shock response (HSR) generally plays a major role in sustaining protein homeostasis. In *Escherichia coli,* the activity and amount of the dedicated transcription factor σ^32^ transiently increase upon heat shock. The initial induction is followed by chaperone-mediated negative feedback to inactivate and degrade σ^32^. Previous work reported that signal recognition particle (SRP)-dependent targeting of σ^32^ to the membrane is essential for feedback control, though how SRP recognizes σ^32^ remained unknown. Extensive photo- and disulfide cross-linking studies *in vivo* now reveal that the highly conserved regulatory region of σ^32^ that lacks a consecutive hydrophobic stretch interacts with the signal peptide-binding site of Ffh (the protein subunit of SRP). Importantly, the σ^32^–Ffh interaction observed was significantly affected by mutations in this region that compromise the feedback regulation, but not by deleting the DnaK/DnaJ chaperones. Homeostatic regulation of HSR thus requires a novel type of SRP recognition mechanism.

The heat shock response (HSR) is a ubiquitous cellular strategy for coping with damaged proteins and maintaining homeostasis by ensuring appropriate expression levels of heat shock proteins (HSPs)^1^. Upon exposure to heat or other stressors, cells undergo rapid and transient induction of HSPs, such as chaperones and proteases, which aid in protein folding or degradation, thereby protecting cells from the stress. Not surprisingly, the HSR requires complex regulatory circuits to meet the needs of various cell types, organisms, and environments. In *Escherichia coli* and other bacteria, σ^32^, the *rpoH* gene product, directs RNA polymerase to promote transcription of a set of HSP genes^2^,^3^,^4^,^5^,^6^. σ^32^ is extremely unstable, and it is normally present at very low levels. When the cell is exposed to heat stress, the activity and level of σ^32^ rapidly increase, due to both elevated translation of *rpoH* mRNA^7^,^8^,^9^,^10^ and transient stabilization of the σ^32^ protein^11^,^12^,^13^. This induction phase is soon followed by the recovery (adaptive) phase, in which the activity/level of σ^32^ gradually decreases to reach a new steady-state. The latter mode of regulation, known as negative feedback control, is mediated by a set of conserved chaperones, including DnaK/DnaJ and proteases that accumulate during induction phase; however, the detailed mechanisms remain unknown, even in *E. coli*, the best-studied bacterial system.

Degradation of σ^32^ is primarily mediated by the essential membrane-localized protease FtsH^14^,^15^. DnaK/DnaJ and GroEL/GroES chaperones can bind σ^32^ directly and promote its degradation *in vivo*^16^,^17^,^18^,^19^,^20^, but this has not been recapitulated *in vitro*^21^. Extensive work on a class of σ^32^ mutants with altered stability and feedback control (dysregulation mutants), identified the homeostatic control region (a segment of Leu-47 to Leu-55 in region 2.1) of σ^32^ that is important for regulating both stability and activity of σ^32^ 22,23,24, although its actual role is not known. Moreover, contrary to the expectations based on *in vivo* results, purified σ^32^ with a strong dysregulation mutation (I54N) exhibits almost normal binding to the chaperones and wild-type sensitivity to inhibition by the chaperones when tested in an *in vitro* transcription system, suggesting that additional factors are involved in σ^32^ regulation^24^. A subsequent search for the missing link led to the finding that signal recognition particle (SRP), which consists of the Ffh protein and 4.5 S RNA, SRP receptor (SR: FtsY), and the SecYEG translocon play essential roles in both chaperone-mediated inactivation and FtsH-mediated degradation of σ^32^ 25. This unexpected finding not only revealed a new regulatory pathway for σ^32^-mediated HSR, but also explained how damage to the SRP pathway, as well as cytoplasmic protein damage, can induce HSR. Moreover, this observation suggested that protein-folding states in the cytoplasm and those in the inner membrane (IM) are integrated and/or coordinated.

In protein transportation to the inner membrane (IM) by the SRP pathway, the M domain of Ffh binds a hydrophobic signal peptide (SP) or a transmembrane segment of the membrane proteins during (or just after) translation on the ribosome (Fig. 1, left)^26^. The nascent chain is targeted to the SecYEG translocon through the SRP–SR interaction, and inserted into the lipid bilayer via the SecY polypeptide–conducting channel and subsequently its lateral gate^27^. Recent crystal structures of an Ffh homolog in complex with an SP have revealed diverse modes of Ffh–SP interactions^28^,^29^,^30^.

Full-length σ^32^ can bind to the M domain of Ffh *in vitro*^25^. Also, analysis of σ^32^–PhoA fusion proteins suggested that the N-terminal part of σ^32^ carries a sequence that can bring the PhoA mature sequence to the membrane-proximal region of the cell in an SRP-dependent manner^25^. Nonetheless, σ^32^ does not contain a stretch that is sufficiently hydrophobic to serve as a typical signal sequence or transmembrane segment, and it was unclear whether the SP-binding site of Ffh is actually involved in the binding of σ^32^. Thus, the mechanism by which Ffh recognizes σ^32^ remained as an intriguing problem to be addressed from the standpoints of both SRP function and heat shock regulation.

In this study, we used *in vivo* cross-linking approaches^31^,^32^,^33^,^34^ to demonstrate that the homeostatic control region of σ^32^ directly interacts with the SP-binding site in the M domain of Ffh and that this interaction is intimately involved in feedback control of σ^32^ (Fig. 1, right). Although the region 2.1 of σ^32^ also interacts with DnaK/DnaJ and other chaperones, the Ffh–σ^32^ interaction revealed by this work does not depend on these chaperones.

## Results

### Ffh binds to the homeostatic control region of σ^32^ *in vivo*

**Figure 2:**
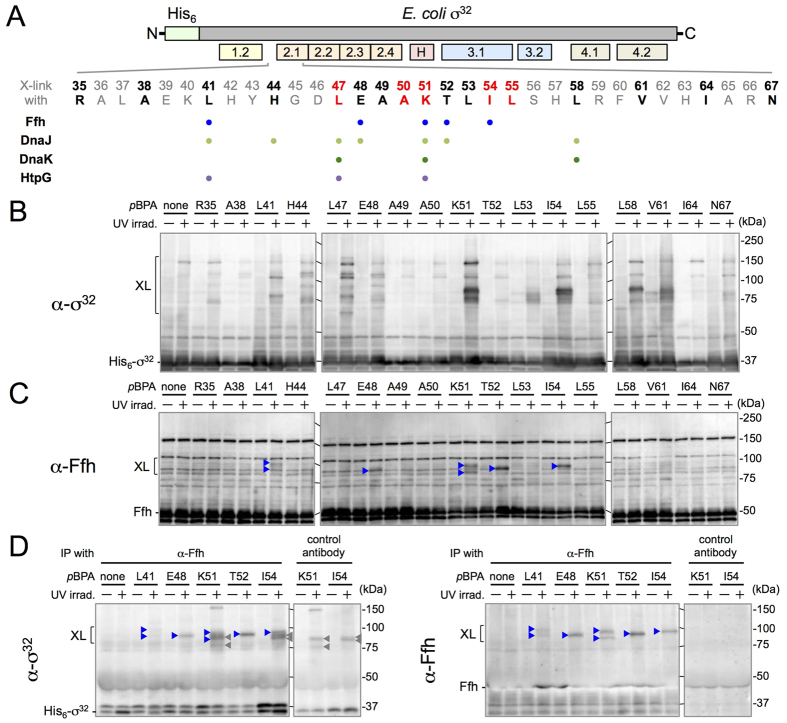
The homeostatic control region of σ^32^ binds to Ffh *in vivo*. (**A**) The region of σ^32^ examined (top) and a summary of the cross-linking studies (below). The homeostatic control region of σ^32^ is located within the regulatory region, region 2.1, conserved among all σ factors. The residues individually mutated to an *amber* codon are shown in bold letters, and those involved in the known dysregulation mutations are shown in red. Positions where cross-linking with Ffh, DnaJ, DnaK, and HtpG was detected clearly and reproducibly are indicated by colored dots. (**B**,**C**) Analysis of cross-linked products by SDS-PAGE and immunoblotting with anti-σ^32^ (**B**) or anti-Ffh (**C**) antibodies. Cells of CAG48238/pEVOL-pBpF/pTTQ18-*his*_*6*_*-rpoH(amb)* were grown at 30 °C in L-medium supplemented with 0.02% arabinose and 1 mM *p*BPA, induced to express His_6_-σ^32^*p*BPA with 1 mM IPTG for 1 h, and UV-irradiated. A strain producing wild type (WT) σ^32^ (shown here as “*p*BPA none”) served as a control (left lane). Total cellular proteins were acid-precipitated and analyzed by 7.5% SDS-PAGE followed by immunoblotting. XL indicates cross-linked products. (**D**) Immunoprecipitation (IP) of cross-linked products with anti-σ^32^ or anti-Ffh antibodies. Extracts of sonically disrupted UV-irradiated cells were subjected to IP with anti-Ffh or control antibodies. Precipitates were analyzed by 7.5% SDS-PAGE followed by immunoblotting with anti-σ^32^ or anti-Ffh antibodies. Blue arrowheads in (**C,D**) indicate the σ^32^–Ffh cross-linked products obtained by anti-σ^32^ or anti-Ffh immunoblotting. Gray arrowheads indicate proteins non-specifically precipitated by the control (SecA6-peptide)^46^ or other antibodies. In (**D**) non-cross-linked Ffh was hardly detected due to the overlapping IgG heavy chains used for immunoprecipitation.

To search for proteins that interact with the homeostatic control region of σ^32^, we employed the *in vivo* photo-cross-linking approach. For this purpose, we introduced a non-natural, photo-reactive amino acid, *p*-benzoyl-L-phenylalanine (*p*BPA), into each of the 17 positions (Arg-35 to Asn-67; see Fig. 2A) in and around the homeostatic control region of N-terminally His_6_-tagged σ^32^ (His_6_-σ^32^); we accomplished these substitutions by *amber* suppression using the laboratory evolved *Methanocaldococcus jannaschii* aminoacyl-tRNA synthetase/suppressor tRNA pair^35^. While most of the His_6_-σ^32^ variants containing *p*BPA (His_6_-σ^32^*p*BPA) exhibited accumulation levels comparable to that of WT His_6_-σ^32^, some (A38, A49 and I64*p*BPA variants) accumulated at much lower levels presumably due to instability, although they all showed similar σ^32^ activities, as determined by LacZ expression from the reporter (P_*htpG*_*-lacZ*) (Supplementary Fig. S1A,B).

Following UV irradiation of cells expressing His_6_-σ^32^*p*BPA, a number of the σ^32^ variants migrated as multiple protein bands with higher apparent molecular masses when analyzed by SDS-PAGE and anti-σ^32^ immunoblotting (Fig. 2B, indicated by XL). Immunoblotting with anti-Ffh antibodies clearly detected putative σ^32^–Ffh cross-linked products at four positions (E48, K51, T52 and I54); weaker bands for putative cross-linked products were also detected at position of L41 (Fig. 2C). We should note that the bands that correspond to σ^32^–Ffh cross-linked products were not directly detected by anti-σ^32^ antibodies due to high backgrounds (Fig. 2B), but became detectable after prior purification by immunoprecipitation with anti-Ffh antibodies (Fig. 2D). These results extend our previous result that showed cross-linking of His_6_-σ^32^T52*p*BPA to Ffh, and clearly demonstrate that Ffh directly interacts with σ^32^ at several positions in the homeostatic control region *in vivo*.

### Chaperones DnaK, DnaJ, and HtpG also interact with the homeostatic control region of σ^32^

Generation of multiple cross-linked products at several positions (including Leu-47 and Lys-51) (Fig. 2B) suggested that protein factors other than Ffh also interacted with the homeostatic control region and its vicinity of σ^32^. To identify these factors, we purified the putative cross-linked products and analyzed them individually by mass spectrometry. Initially, we analyzed the cross-linked products obtained with two His_6_-σ^32^*p*BPA proteins, His_6_-σ^32^L47*p*BPA and His_6_-σ^32^K51*p*BPA, both of which yielded multiple cross-linked products, whereas only His_6_-σ^32^K51*p*BPA was cross-linked to Ffh. To improve the efficiency of His-tag affinity purification, we used a longer tag (His_10_); this modification barely affected the cross-linking profiles (Supplementary Fig. S2A,B).

The cross-linked products were purified under SDS-denaturing conditions, separated by SDS-PAGE, and subjected to mass spectrometry analysis (Supplementary Fig. S2C,D). Consistent with the results of immunoblotting (Fig. 2B,C), Ffh fragments were detected from the His_10_-σ^32^K51*p*BPA sample, but not from the His_10_-σ^32^L47*p*BPA sample. In addition, amino acid sequences of chaperones, DnaK, DnaJ, and HtpG, were found in the products formed at both positions (Supplementary Fig. S2C,D). Cross-linking of His_6_-σ^32^*p*BPA with these chaperones at all the 17 positions was then examined by immunoblotting using appropriate anti-chaperone antibodies. Cross-linking with these chaperones was detected at seven positions, including L47 and K51 (Supplementary Fig. S3A–C). These results demonstrate that DnaK, DnaJ, and HtpG interact rather promiscuously with the homeostatic control region of σ^32^, as summarized in Fig. 2A.

### σ^32^ mutations that compromise feedback control affect the interaction between σ^32^ and Ffh

**Figure 3:**
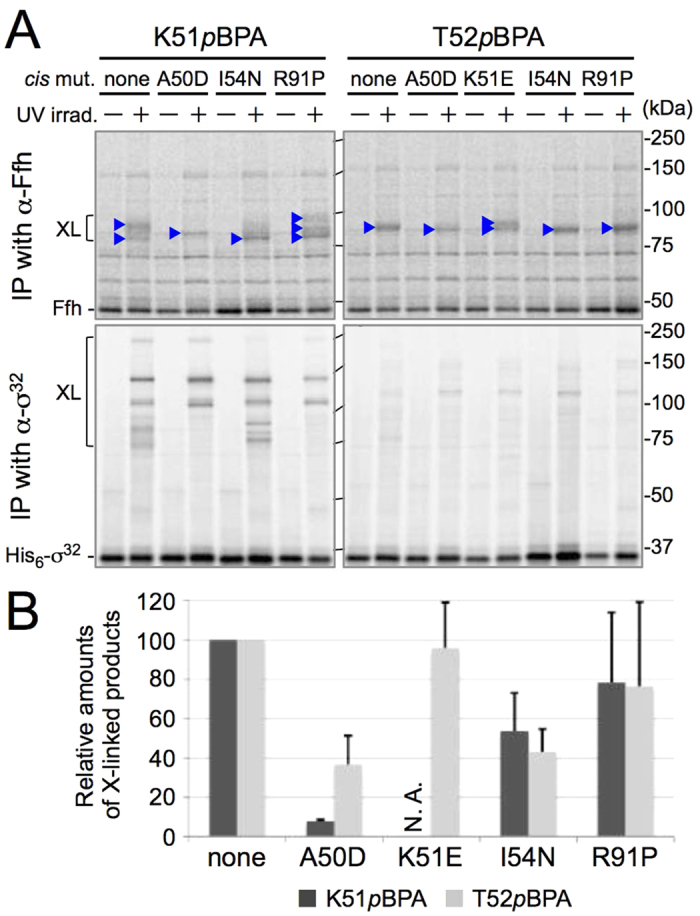
σ^32^ dysregulation mutations affect the mode and extent of the σ^32^–Ffh interaction. (**A**) *In vivo* photo-cross-linking using [^35^S]Met-pulse-labeled His_6_-σ^32^*p*BPA proteins. Plasmid pTTQ18-*his*_*6*_*-rpoH(K51amb)* or pTTQ18-*his*_*6*_*-rpoH(T52amb),* with or without additional dysregulation mutation, was transformed into CAG48238/pEVOL-pBpF/pRM83-*ffh+ffs.* Cells of the resulting strains were grown at 30 °C in M9-based medium with 1 mM *p*BPA, induced to express His_6_-σ^32^*p*BPA with 1 mM IPTG for 6 min, and labeled with [^35^S]Met for 1 min. Following UV irradiation, total cellular proteins were immunoprecipitated (IP) with anti-σ^32^ or anti-Ffh antibodies, and immunocomplexes were separated by 7.5% SDS-PAGE followed by phosphorimaging. (**B**) Quantification of His_6_-σ^32^–Ffh cross-linked products. The ratio of the sum of intensities of His_6_-σ^32^–Ffh cross-linked products to that of unirradiated His_6_-σ^32^ is shown (the values for His_6_-σ^32^*p*BPA carrying no dysregulation mutation were set to 100). Three independent experiments were performed, and mean values are shown along with standard deviations.

To further investigate the significance of the observed σ^32^–Ffh interaction in the negative feedback control of σ^32^, we tested the effects of the previously characterized dysregulation mutations^24^, A50D, K51E, I54N, and R91P, on the σ^32^–Ffh cross-linking. These mutations, when introduced in *cis*, stabilized both His_6_-σ^32^K51*p*BPA and His_6_-σ^32^T52*p*BPA as expected, resulting in elevated and variable levels of protein accumulation (Supplementary Fig. S4A,B). This made it difficult to accurately evaluate the effects of these mutations on σ^32^–Ffh cross-linking efficiencies. Because the synthesis rates of His_6_-σ^32^*p*BPA variants with or without the dysregulation mutations were nearly equal, we performed cross-linking using cells pulse-labeled with radioactive methionine (Fig. 3A). Cells expressing the His_6_-σ^32^K51*p*BPA or His_6_-σ^32^T52*p*BPA protein (without dysregulation mutation) were first labeled with [^35^S]Met, UV-irradiated, and subjected to immunoprecipitation with anti-σ^32^ or anti-Ffh antibodies. The results showed that the profiles of the radioactive cross-linking products were very similar to those detected by immunoblotting (compare Figs 3A and 2B). We then examined His_6_-σ^32^K51*p*BPA and His_6_-σ^32^T52*p*BPA having each of the σ^32^ dysregulation mutations. Remarkably, these mutations altered the profiles and/or amounts of σ^32^–Ffh cross-linking products (Fig. 3A). Whereas the parental His_6_-σ^32^ T52*p*BPA generated a single cross-linked product of approximately 85 kDa, the same protein with the A50D, I54N, or R91P mutation generated a single cross-linked product of slightly higher mobility. The same protein with the K51E mutation generated two cross-linked products, one migrating faster and another migrating slower. Moreover, the two strong mutations, A50D and I54N, caused clear reduction in the relative amounts of cross-linked products, whereas only little effects were observed with weak mutations, K51E and R91P (Fig. 3B). In the case of His_6_-σ^32^K51*p*BPA, the parental protein generated two cross-linked products of approximately 80 and 90 kDa, whereas the A50D, I54N, and R91P mutations significantly altered the mobility, the number, and/or the relative amounts of cross-linked products (Fig. 3A,B).

Cross-linking of two proteins reflects spatial proximity but not necessarily functional interactions between them. The observed changes in the cross-linking profiles by the σ^32^ dysregulation mutations suggest that each of the mutations caused a shift in the selection of cross-linking partner residue of Ffh. While the altered cross-linking profiles could have resulted from changes in the orientation of the photo-reactive side chain of *p*BPA, which was induced by the dysregulation mutations introduced into nearby positions in the primary structure, characteristic alterations in the number and mobility of the cross-linked products were also observed for the R91P mutation, which affects a position distant from that of *p*BPA in the primary structure but predicted to be nearby in the folded structure^24^. It should also be noted that, while the previous gel filtration experiments with purified proteins demonstrated that the I54N mutation apparently abolished the σ^32^–Ffh interaction, the current *in vivo* cross-linking results showed that the interaction was only moderately affected by the same mutation. This difference may be ascribed to different sensitivities of these methods; *in vivo* cross-linking could enable detection of weaker and/or transient interactions that would be missed by gel-filtration assays. In addition, the σ^32^ *cis* mutations exerted parallel effects on the negative regulation of σ^32^ and on the cross-linking of σ^32^ with Ffh; the stronger mutations exerted more severe effects. These results strongly implicate that the observed changes in the amounts and profiles of the cross-linking products are at least partly ascribable to some changes in the σ^32^–Ffh binding interfaces and that the σ^32^–Ffh cross-linking indeed represents functional client–machinery interactions involved in the homeostatic control of σ^32^.

### DnaK/DnaJ chaperones are not required for *in vivo* interaction of Ffh with the homeostatic control region of σ^32^

Previous studies established that the DnaK/DnaJ chaperone system is involved in the negative feedback control of σ^32^ 16,17,18,19,20,21. Because DnaK and Dn aJ directly interact with σ^32^ 17,18, we considered the possibility that these chaperones induce conformational changes in σ^32^ to facilitate its recognition by Ffh. We addressed this possibility by using a *dnaKJ* deletion strain as a host for σ^32^–Ffh photo-cross-linking experiments involving the His_6_-σ^32^ K51*p*BPA and His_6_-σ^32^ T52*p*BPA proteins. Anti-Ffh immunoblotting revealed that the absence of DnaK/DnaJ chaperones caused no distinct alteration in the profiles and the levels of σ^32^–Ffh cross-linked products (Supplementary Fig. S5A,B). On the other hand, anti-σ^32^ immunoblotting analysis revealed that cross-linked products of about 150 kDa and 75 kDa disappeared from blots of His_6_-σ^32^ K51*p*BPA and His_6_-σ^32^T52*p*BPA, respectively (Supplementary Fig. S5A, lower panel). The corresponding bands were detected specifically with anti-DnaK and anti-DnaJ antibodies when the wild-type host was used, indicating that they represent the σ^32^–DnaK and σ^32^–DnaJ cross-linked products, respectively (Supplementary Fig. S3). These results show that the DnaK/DnaJ chaperones do bind to σ^32^, but they play no essential role in the interaction of Ffh with the homeostatic control region of σ^32^ observed in this study, consistent with our previous result that purified σ^32^ and SRP interact in the absence of DnaK/DnaJ^25^.

### Ffh uses its signal peptide-binding site to bind the homeostatic control region of σ^32^

**Figure 4:**
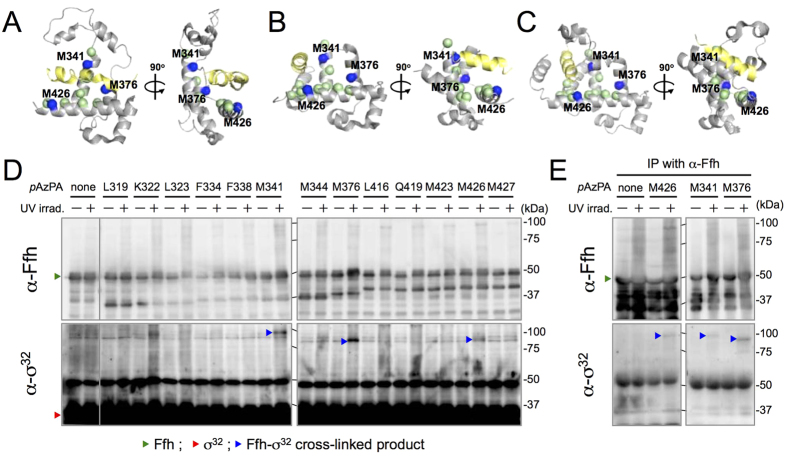
σ^32^ interacts with the SP–binding site of Ffh *in vivo.* (**A**–**C**) Crystal structures of *Ss*SRP54–SP (PDB:3kl4; **A**)^28^, *Mj*SRP54–SP (PDB:3ndb; **B**)^29^, and *Mj*SRP54 M–SRP19–SRP RNA (PDB:4xco; **C**)^30^ in complex with SP. Only the M domain of each Ffh homolog (SRP54) is shown with SP (in yellow). The positions corresponding to those in *E. coli* Ffh (see Supplementary Fig. S6) where *p*AzPA was incorporated are indicated by spheres: the positions where cross-linking with σ^32^ was detected are colored in blue (the corresponding residues of *E. coli* Ffh are indicated on the models), and the others are in light green. (**D**) Immunoblotting analysis of *in vivo* photo cross-linking using Ffh*p*AzPA variant proteins. Cells of CAG48373 (Δ*ftsH sfhC21*)/pEVOL-pAzF/pTTQ18-*ffh(amb)+ffs* were grown at 30 °C in L-medium supplemented with 0.02% arabinose and 1 mM *p*AzPA, induced to express Ffh*p*AzPA with 1 mM IPTG for 1 h, and UV-irradiated as indicated. Total cellular proteins were analyzed by 7.5% SDS-PAGE and immunoblotted with anti-Ffh and anti-σ^32^ antibodies. (**E**) Immunoprecipitation (IP) of cross-linked products with anti-Ffh antibodies. Total cellular proteins of UV-irradiated cells were precipitated with anti-Ffh antibodies, and analyzed by 7.5% SDS-PAGE followed by immunoblotting with anti-σ^32^ and anti-Ffh antibodies.

We next selected Ffh residues as a site of photo-cross-linker introduction in attempts to identify the residue(s) of Ffh that interact(s) with σ^32^ *in vivo*. The recently reported crystal structures of the Ffh homologs from *Sulfolobus solfataricus* (Fig. 4A)^28^ and *M. jannaschii* (Fig. 4B,C)^29^,^30^, each in complex with a signal peptide (SP), indicate that in each of these well conserved structures (Supplementary Fig. S6), the SP binds to the M domain of SRP54 in a distinct manner. Based on these structures, we selected 13 residues encompassing the possible SP-binding sites in the *E. coli* Ffh M domain as the sites of photo-cross-linker introduction. We used *p*-azido-L-phenylalanine (*p*AzPA)^35^ instead of *p*BPA to avoid cross-linking interference by nearby methionine residues in the M domain^36^. We confirmed that full-length, *p*AzPA-containing Ffh variants (Ffh*p*AzPA) accumulated at similar levels across the different constructs (Supplementary Fig. S7A). All the Ffh*p*AzPA variants tested supported growth of cells depleted for wild-type Ffh, indicating that they were functional (Supplementary Fig. S7B).

To facilitate detection of Ffh–σ^32^ cross-linked products, we used as a host an FtsH-deletion strain that allowed increased levels of σ^32^ accumulation. Anti-σ^32^ immunoblotting analysis revealed that bands of ~80–100 kDa were generated in a UV-irradiation dependent manner when *p*AzPA was introduced in place of Met-341, Met-376, or Met-426 of Ffh, whereas no such bands were detected at other positions or with wild-type Ffh (no *p*AzPA incorporated) (Fig. 4D). The UV- and *p*AzPA-dependent generation of these bands, although less marked at positions 341 and 426, strongly suggests that they represent products of cross-linking between Ffh and σ^32^. These bands were not detectable by anti-Ffh immunoblotting, probably due to high background caused by overexpression of Ffh. However, these bands could be detected by anti-σ^32^ immunoblotting if preceded by immunoisolation with anti-Ffh antibodies, but not with control antibodies (Fig. 4E and Supplementary Fig. S8), indicating that they all contained both Ffh and the σ^32^ polypeptides. By contrast, no cross-linking of FfhM376*p*AzPA with σ^E^, another alternative sigma factor involved in the extracytoplasmic stress response^6^, was observed (Supplementary Fig. S9). These results suggest that σ^32^ directly and specifically interacts with the SP-binding region of Ffh M domain *in vivo.*

The sites of σ^32^–Ffh interaction and the role of SP-binding site of Ffh were investigated further by means of disulfide-cross-linking experiments, featuring site-specifically introduced Cys residues in these proteins. As a σ^32^ derivative, we constructed His_10_-σ^32^T52C by replacing Thr-52 with Cys (note that WT-His_10_-σ^32^ has no intrinsic Cys residue, and that Thr-52 is the position where stable photo-cross-linking with Ffh was consistently observed) (Fig. 2). The mutant protein accumulated normally with unaltered activity (Supplementary Fig. S10A,B). As single Cys Ffh derivatives, we first constructed Cys-less Ffh (C406S), into which Cys was introduced at each of three positions, Met-341, Met-376 and Met-426, where we observed photo-cross-linking with σ^32^ (Fig. 4). None of the cysteine substitutions affected protein levels or activities (Supplementary Fig. S10C,D).

**Figure 5:**
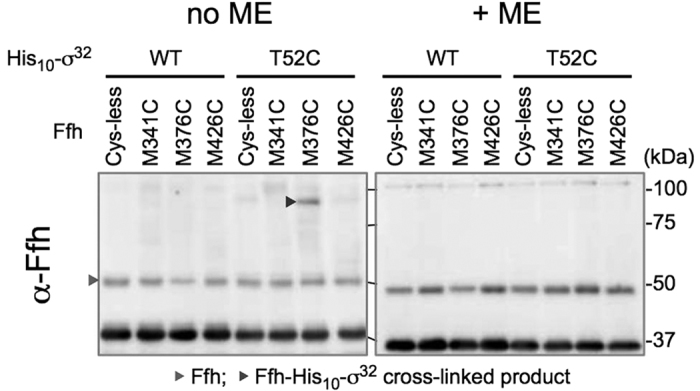
Disulfide cross-linking between the homeostatic control region of σ^32^ and the SP-binding region of Ffh. Cells of WAM121 (Δ*ffh1*::*kan* P_*ara*_*-ffh*) carrying a combination of plasmids encoding Cys-less or single Cys-derivative of His_10_-σ^32^ and Ffh as indicated were grown in L-medium and induced to express His_10_-σ^32^ Cys and Ffh Cys with 1 mM IPTG for 1 h. Cells were treated with Cu^2+^(phenanthroline)_3_ at 37 °C for 5 min. After quenching the oxidant and blocking free thiol groups, extracts of sonically disrupted cells were subjected to TALON affinity purification. The purified proteins were treated with or without 2-mercaptoethanol (ME) and analyzed by 7.5% SDS-PAGE and immunoblotting with anti-Ffh antibodies.

Cells expressing a combination of His_10_-σ^32^T52C and one of the single-Cys Ffh derivatives were treated with an oxidant, Cu^2+^(phenanthroline)_3_, to induce any possible disulfide bond formation. After quenching the oxidant, total cellular proteins were subjected to His-tag affinity isolation. Samples were then separated by SDS-PAGE in the presence or the absence of 2-mercaptoethanol (ME) and analyzed by anti-Ffh immunoblotting. The oxidant treatment generated a band of about 90 kDa for the combination of His_10_-σ^32^T52C and FfhM376C but not for the others (Fig. 5, no ME). The 90 kDa band disappeared when the sample had been treated with ME (Fig. 5, +ME). Its generation depended on the simultaneous presence of the Cys residues in His_10_-σ^32^ and Ffh. These results indicate that the residue 52 of σ^32^ and residue 376 of Ffh are within a distance that allows intermolecular formation of a disulfide bond when they are replaced with Cys. Taken together, our results demonstrate that Ffh directly binds the homeostatic control region of σ^32^ at the SP-binding site, such that the residue 376 of Ffh is in close proximity to the residue 52 of bound σ^32^ (see Fig. 4A–C and Discussion for possible modes of σ^32^–Ffh interaction).

## Discussion

Our previous work revealed the unexpected involvement of the SRP-mediated membrane-targeting pathway in the HSR regulatory circuit governed by the transcription factor σ^32^, which connects cytoplasmic and IM proteostasis in *E. coli*^25^. Although we showed that SRP can directly bind σ^32^ *in vivo* and *in vitro*, it remained unclear how SRP and σ^32^ interact with each other. We investigated this issue using three variations of *in vivo* cross-linking techniques. The results reveal that the homeostatic control region of σ^32^ directly interacts with not only the DnaK, DnaJ, and HtpG chaperones but also Ffh at multiple positions, suggesting that this region of σ^32^ serves as a hub for molecular interactions involving SRP and chaperones for the homeostatic control of HSR (Fig. 2). The finding that several σ^32^ dysregulation mutations affect the extent and profile of cross-linking between σ^32^ and Ffh (Fig. 3) reinforces the notion that the observed σ^32^–Ffh interaction is important for the homeostatic control of σ^32^. In sharp contrast, this interaction was detected in the total absence of DnaK/DnaJ chaperones (Supplementary Fig. S5), indicating that these chaperones are not essential for the σ^32^–Ffh interaction observed.

The results of the photo- and disulfide-cross-linking experiments demonstrated that the homeostatic control region of σ^32^ directly and specifically interacts with the SP binding region in the M domain of Ffh *in vivo*. This observation is consistent with and complements the previous results of far western blotting analysis showing that purified σ^32^ binds to the M domain fragment of Ffh *in vitro*^25^. According to the published crystal structures of SRP54(Ffh)–SP complexes from archaeal species, the M domain of SRP54 invariably interacts with SP, but in slightly different manners (Fig. 4A–C)^28^,^29^,^30^. We detected photo cross-linking of σ^32^ at three (Met-341, Met-376, and Met-426) out of 13 positions tested in the *E. coli* Ffh M domain (Fig. 4D). In addition, Cys at position 376 of Ffh was disulfide-bonded with Cys at position 52 in the homeostatic control region of σ^32^. Among the three positions where we detected cross-linking, two (Met-341 and Met-426) are located near the bound SP in all the reported crystal structures (cf. Fig. 4A–C), whereas the other (Met-376) is located near SP only in the *S. solfataricus* SRP54–SP complex (Fig. 4A). These results and the structural information suggest that *E. coli* Ffh binds σ^32^ at the SP binding site, which could be shared by transmembrane segments of IM proteins, in a manner similar to the *S. solfataricus* SRP54–SP interaction. Such substrate-binding sites shared by IM proteins and σ^32^ would endow the cell with a robust and versatile capacity to respond to changes in protein folding status in the cytoplasm and IM. Thus, the dynamic change in the extent of interaction between SRP and σ^32^ may provide the basis for effective control of proteostasis during normal growth, as well as under stress.

Whereas SRP generally binds to hydrophobic polypeptide sequences, mostly transmembrane segments in bacteria^37^, the homeostatic control region of σ^32^ contains no consecutive array of hydrophobic residues. Sequence-based secondary structure prediction suggests that the homeostatic control region of σ^32^ forms an amphipathic α-helix (Supplementary Fig. S11A,B). This amphipathic helix might provide a hydrophobic surface for Ffh binding (Supplementary Fig. S11B). Consistent with this hypothesis, the strong dysregulation mutations A50D and I54N, which introduce a charged and a hydrophilic residue, respectively, into the hydrophobic surface of the predicted amphipathic helix, strongly affected Ffh binding (Fig. 3). In the *S. solfataricus* SRP54–SP complex, SP binds to the M domain in a partially unraveled configuration (Fig. 4A). The σ^32^ homeostatic control region might also be partially deformed to fit the SP binding site. Moreover, σ^32^K51*p*BPA generated two cross-linked products with Ffh (Fig. 3A), indicating that this position can contact two different sites in Ffh. This might reflect either flexibility in the interaction between the SP binding site and the σ^32^ homeostatic control region or the involvement of functionally distinct modes of interactions.

Our previous *in vitro* experiments using purified components showed that SRP can bind to folded σ^32^ 25, demonstrating that SRP can recognize σ^32^ post-translationally. This study also supports the notion that σ^32^ interacts with SRP post-translationally *in vivo*, as full-length σ^32^ was able to cross-link with Ffh. Several earlier studies have suggested that SRP can target substrate proteins to the membrane post-translationally^38^,^39^. Post-translational SRP recognition of folded σ^32^ may be necessary to target accumulated σ^32^ to FtsH-mediated degradation during the recovery phase of HSR. On the other hand, σ^32^ may also interact with SRP co-translationally to allow targeting of its newly synthesized molecule to the membrane, where it is eventually inactivated and degraded. SRP-mediated co-translational membrane targeting of σ^32^ may well facilitate its rapid turnover under normal growth conditions. These possibilities should be addressed in future analyses.

In addition to Ffh, the DnaK, DnaJ, and HtpG chaperones interacted with the homeostatic control region of σ^32^. This finding is consistent with previous reports that DnaJ can bind to region 2.1 *in vitro*^40^,^41^. By contrast, DnaK has been suggested to interact *in vitro* with a region (around residues 198–200) C-terminal to the homeostatic control region^40^. Our results described here indicate that DnaK interacts with this control region with significant affinity *in vivo*. Indeed, a recent study suggested the existence of multiple DnaK binding sites in σ^32^ 42. If SRP and DnaK/DnaJ chaperones act at distinct steps in the σ^32^ control circuit, these factors might bind to the homeostatic control region in a sequential manner, although we currently do not know whether SRP and DnaK/DnaJ can bind to the same σ^32^ molecule simultaneously or whether their binding is mutually exclusive. *In vitro* experiments with defined components will be needed to clarify these points. Although our results suggest that HtpG also interacts with σ^32^, this chaperone does not appear to play a critical role in the regulation of σ^32^, because a loss or an overexpression of HtpG has very little effect on the σ^32^ activity 16. Because HtpG can assist the DnaK/DnaJ chaperones 43, it could play a limited or auxiliary role in the σ^32^ regulation by modulating DnaK/DnaJ functions. Clearly, further work is needed to elucidate the significance of the observed binding between chaperones and the homeostatic control region in σ^32^ regulation.

It is intriguing that SRP can interact with a non-canonical substrate protein like σ^32^, which has no typical SP or transmembrane segment, to regulate its localization and function. Recent work showed that mammalian SRP also plays a critical role in the regulation of the unfolded protein response by promoting the Ire1α-mediated splicing of XBP1u mRNA^44^. In this case, SRP recognizes the ribosome-associated nascent XBP1u polypeptide to target it to the Ire1α/Sec61 complex on the ER membrane together with the XBP1u mRNA also in complex with the ribosome. Taken together with our findings in *E. coli*, these observations suggest that SRP plays an important novel role in the peripheral association of some proteins by assisting their targeting to the membrane. Like the homeostatic control region of σ^32^, the moderate hydrophobic region (HR2) required for SRP-mediated membrane targeting of XBP1u is predicted to form an amphipathic helix (Supplementary Fig. S11C). Thus, SRP, either prokaryotic or eukaryotic, could be able to recognize a certain class of amphipathic helices. It is important to study whether this new function of SRP requires any other properties such as the primary sequences, higher order structures, and separate *cis*-elements in the non-canonical substrates, as well as any other cellular components (*trans* factors). As shown here, the techniques of *in vivo* cross-linking are useful to capture transient substrates as well as more stable reaction partners of the system. Further systematic analysis targeted to Ffh may prove useful for identifying and analyzing some additional proteins that may be subject to and involved in the SRP-mediated localization/regulation. Such studies may reveal unexpected links between known cellular events and membrane functions.

In the case of HSR regulation in *E. coli*, it remains to be asked how this targeting event is productively coupled with subsequent events of feedback regulation, in which proteolysis and chaperone binding may be involved in a well-balanced fashion to poise the cell for forthcoming stresses. The new SRP pathway may contribute to the bidirectional regulation by allowing dual localization of σ^32^, a key regulator of bacterial proteostasis.

## Methods

### Bacterial Strains

*Escherichia coli* K12 strains used in this study are listed in Supplementary Table S1. CAG48238, a derivative of MG1655^24^ carrying σ^32^-dependent reporter P_*htpG*_*-lacZ*, was used as a wild-type strain. RM591 was a derivative of CAG48238 carrying a Δ*dnaKJ*::*kan* marker (a gift from C. A. Gross and B. Lim, University of California), but without the *cat* marker. WAM121 (Δ*ffh1*::*kan* P_*ara*_*-ffh*)^45^ was a generous gift from G. J. Phillips (Iowa State University).

### Plasmids and Primers

Plasmids and primers used in this study are listed in Supplementary Table S2 and S3, respectively, and details of the plasmid construction are described in Supplementary information.

### Antibodies

Penta-His HRP conjugate was purchased from QIAGEN (Hilden), anti-σ^32^ antibody used for immunoprecipitation (RNA pol σH 3RH3) from Santa Cruz Biotechnology, and anti-σ^E^ antibody (Sigma E antibody) from MyBioSource. The control antibody used in Fig. 2D and Supplementary Fig. S8 was prepared from a rabbit immunized with a SecA peptide but recognized SecA very poorly^46^. Other antibodies used were kindly provided by various sources; anti-σ^32^ used for immunoblotting from M. Kanemori (Kanazawa University), anti-Ffh from C. A. Gross (University of California), anti-DnaK from R. McMacken (Johns Hopkins Bloomberg School of Public Health), anti-DnaJ from A. H. Becker (University of Heidelberg), and anti-HtpG from F. Motojima (Toyama Prefectural University).

### *In vivo* Photo-Cross-Linking

*In vivo* photo-cross-linking experiments with *p*BPA-introduced His_6_-σ^32^ (His_6_-σ^32^*p*BPA) were carried out essentially as described previously^25^. For immunoblotting analysis, cells of CAG48238 carrying pEVOL-pBpF and one of the pTTQ18-*his*_*6*_*-rpoH(amb)* plasmids were grown at 30 °C in L-medium supplemented with 0.02% arabinose and 1 mM *p*BPA to an early log phase (0.2 TAITEC units), induced with 1 mM IPTG to express His_6_-σ^32^*p*BPA for 1 h, and UV-irradiated at 4 °C for 10 min on a petri dish using B-100 AP UV lamp (365 nm; UVP, LLC.) at a distance of 4 cm. Total cellular proteins were precipitated with 5% trichloroacetic acid (TCA), washed with acetone, and solubilized in SDS sample buffer. Proteins were analyzed by 7.5% Laemmli SDS-PAGE followed by immunoblotting and visualization using ECL^TM^ Western Blotting Detection Reagents or ECL^TM^ Prime Western Blotting Detection Reagents (GE Healthcare) and LAS4000 mini lumino-image analyzer (GE Healthcare). Band intensities were quantified with MultiGauge software (GE Healthcare). For pulse-labeling analysis, cells of strain CAG48238 carrying pEVOL-pBpF, pRM83-*ffh+ffs* and one of the pTTQ18-*his*_*6*_-*rpoH(amb)* derivatives additionally carrying the A50D, K51E, I54N or R91P mutation were grown at 30 °C in M9-medium supplemented with 2 μg/ml thiamine, 0.4% glycerol, 0.2% maltose, 18 amino acids (except Met and Cys; final concentration of 20 μg/mL each), 1 mM *p*BPA until early log phase (0.1 TAITEC units), and induced with 1 mM IPTG to express His_6_-σ^32^*p*BPA for 6 min. Cells were labeled with 370 kBq/ml [^35^S]Met (American Radiolabeled Chemicals) for 1 min. After addition of excess nonradioactive Met and Cys (final conc. 250 μg/ml each), cells were immediately chilled on ice, and UV-irradiated at 4 °C as described above. Total cellular proteins were precipitated with 5% TCA, washed with acetone, and solubilized in buffer containing 50 mM Tris-HCl (pH 8.1), 1% SDS, 1 mM EDTA. Samples with equal radioactivities were subjected to immunoprecipitation using an appropriate antibody essentially as described^47^. Proteins were separated by 7.5% SDS-PAGE, and visualized with BAS1800 phosphoimager (FUJIFILM). Band intensities were quantified with MultiGauge software.

*In vivo* photo-cross-linking experiments using *p*AzPA-introduced Ffh (Ffh*p*AzPA) were carried out as follows. Cells of CAG48373 (Δ*ftsH3*::*kan sfhC21*) carrying pEVOL-pAzF and one of the pTTQ18-*ffh(amb)+ffs* plasmids were grown at 30 °C in L-medium supplemented with L-0.02% arabinose and 1 mM *p*AzPA until early log phase (25 Klett units), and induced with 1 mM IPTG to express Ffh*p*AzPA for 1 h. Cells were collected by centrifugation, suspended in L-medium supplemented with 0.02% arabinose at approximately 4 × 10^8 ^cells/ml, and UV-irradiated at 4 °C for 30 min in a 24-well plate using compact UV lamp 4 W (254 nm; UVP, LLC.) at a distance of 3 cm. Total cellular proteins were precipitated with 5% TCA, washed with acetone, solubilized in SDS sample buffer, and analyzed by 7.5% SDS-PAGE and immunoblotting.

### Purification and Mass Spectrometry Analysis of Cross-linked Products

Cells of CAG48238 carrying pEVOL-pBpF and either pTTQ18-*his*_*10*_*-rpoH(L47amb) or* pTTQ18-*his*_*10*_*-rpoH(K51amb)* were grown at 30 °C in L-medium supplemented with 0.02% arabinose and 1 mM *p*BPA until early log phase (0.2 TAITEC units), and induced with 1 mM IPTG for 1 h. Cells were UV-irradiated as described above, disrupted by sonication in 10 mM Tris-HCl (pH 8.1) at 0 °C. After ultracentrifugation of cell lysates at 100,000 × g for 60 min, 3.8 ml of supernatants were mixed with 1.2 ml of Wash buffer A (50 mM Tris-HCl (pH 8.1), 150 mM NaCl, 0.1% SDS, 0.1 mM EDTA), and incubated with TALON resin (Takara Bio) at room temperature for 2.5 h with rotation. After washing the resin with Wash buffer A, proteins were eluted with wash buffer containing 81 mM EDTA. Eluted proteins were separated on SDS-PAGE and silver stained with Sil-Best Stain One (NACALAI TESQUE). The bands detected after UV radiation were excised and digested in gel with a TPCK-treated bovine trypsin (Worthington Biochemical). Then digest was analyzed by nano liquid chromatography–tandem mass spectrometry (LC-MS/MS) using Q Exactive mass spectrometer (Thermo Fisher Scientific). The peptides were separated using nano ESI spray column (75 μm [ID] × 100 mm [L], NTCC analytical column C18, 3 μm, Nikkyo Technos) with a linear gradient of 0%-35% buffer B (100% acetonitrile and 0.1% formic acid) at a flow rate of 300 nL/min over 10 min (Easy nLC; Thermo Fisher Scientific). The mass spectrometer was operated in the positive-ion mode, and the MS and MS/MS spectra were acquired in a data-dependent TOP10 method. The MS/MS raw data set was searched against the NCBI-nr database using local MASCOT server (version 2.3; Matrix Science Ltd., London, UK). The taxonomy was selected as *Escherichia coli* and the variable modifications were selected as acetyl (protein N-term), deamidated (NQ), formyl (protein N-term), Gln->pyro-Glu (N-term Q) and oxidation (M).

### Immunoprecipitation of σ^32^–Ffh Cross-linked Products

Anti-Ffh immunoprecipitation of cross-linked products of His_6_-σ^32^*p*BPA and Ffh*p*AzPA were carried out as follows. For His_6_-σ^32^*p*BPA-cross-linked products, UV-irradiated cells were suspended in 10 mM Tris-HCl (pH 8.1) and disrupted by sonication at 0 °C. After removal of total membranes by ultracentrifugation, proteins were precipitated with 5% TCA. For Ffh*p*AzPA*-*cross-linked products, UV-irradiated cells were suspended in 10 mM Tris-HCl (pH 8.1), and total cellular proteins were precipitated with 5% TCA. TCA-precipitated proteins were washed with acetone, solubilized in buffer containing 50 mM Tris-HCl (pH 8.1), 1% SDS and 1 mM EDTA and diluted 33-fold with NP40 buffer (50 mM Tris-HCl (pH 8.1), 150 mM NaCl, 1% NP40). After centrifugation, supernatants were incubated with True-Blot anti-Rabbit Ig IP Beads (eBioscience) and either anti-Ffh antibodies or control antibodies at 4 °C for 13 h with rotation. Immunocomplexes were isolated by centrifugation, washed 2 times with wash buffer and once with 10 mM Tris-HCl (pH 8.1), and dissolved in SDS sample buffer. Proteins were separated by 7.5% SDS-PAGE, and analyzed by immunoblotting using appropriate antibodies, TrueBlot anti-Rabbit IgG (eBioscience), and Can Get Signal immunoreaction enhancer solution (TOYOBO) as described previously^25^.

### Disulfide-Cross-linking

Cells of WAM121 carrying pTTQ18-*his*_*10*_*-rpoH* or pTTQ18-*his*_*10*_*-rpoH(T52C)* in addition to one of the pSTD689-*ffh(Cys)+ffs* plasmids were grown overnight at 30 °C in L-medium supplemented with 0.2% arabinose. After washing the cells 3 times with L-medium, they were inoculated into L medium, grown at 30 °C for 3 h and induced with 1 mM IPTG to express His_10_-σ^32^ and Ffh derivatives for 1 h. Cells were washed with 10 mM Tris-HCl (pH 8.1), suspended in 10 mM Tris-HCl (pH 8.1) and treated with 50 μM Cu^2+^(phenanthroline)_3_ at 37 °C for 5 min. The oxidation reaction was terminated by incubation with 2.5 mM neocuproine for 5 min at 37 °C followed by additional incubation with 12.5 mM *N*-ethylmaleimide (NEM) for 10 min at 0 °C. Cells were washed with 10 mM Tris-HCl (pH 8.1), suspended in 10 mM Tris-HCl (pH 8.1) containing 10 mM NEM and disrupted by sonication at 0 °C. After removal of unbroken cells, 2 ml of cell extracts were mixed with 0.5 ml of Wash buffer B (50 mM Tris-HCl (pH 8.1), 150 mM NaCl, 0.1% SDS), and incubated with TALON resin at room temperature for 2.5 h with rotation. After washing the resin with Wash buffer B, proteins were eluted with Wash buffer B containing 81 mM EDTA. Eluted proteins were mixed with 2x SDS-sample buffer with or without 10% β-mercaptoethanol and incubated for 5 min at 98 °C. Purified proteins were analyzed by 7.5% SDS-PAGE followed by anti-Ffh immunoblotting.

## Additional Information

**How to cite this article**: Miyazaki, R. *et al*. A Novel SRP Recognition Sequence in the Homeostatic Control Region of Heat Shock Transcription Factor σ^32^. *Sci. Rep.* **6**, 24147; doi: 10.1038/srep24147 (2016).

## Supplementary Material

Supplementary Information

## Figures and Tables

**Figure 1:**
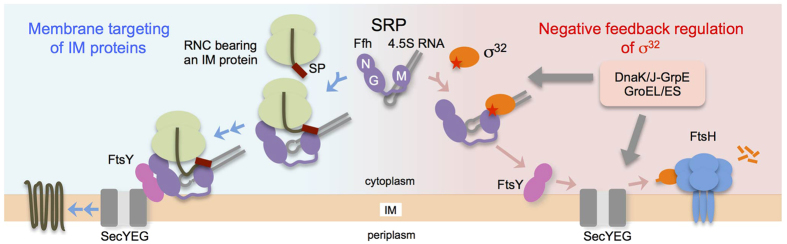
Roles of SRP in membrane protein biogenesis and feedback control of σ^32^. (*left*) Membrane targeting of inner membrane (IM) proteins. The Ffh M domain of SRP interacts with a transmembrane segment (or a signal peptide) of a membrane protein emerging from the ribosomal tunnel. SRP targets the ribosome–nascent chain complex (RNC) to the SecYEG translocon through the interaction between SRP and SR (FtsY). (*right*) Negative feedback control of σ^32^. The homoeostatic control region of σ^32^ (red star) is recognized by the Ffh M domain, and σ^32^ is targeted to SecYEG through the SRP–SR interaction, and degraded by FtsH protease in a chaperone-dependent manner.
