# Supplementary material for: A Novel SRP Recognition Sequence in the Homeostatic Control Region of Heat Shock Transcription Factor σ32
Source: Sci Rep. 2016 Apr 7;6:24147. doi: 10.1038/srep24147 (PMC4823717; doi:10.1038/srep24147)
Supplement: Supplementary Information [file srep24147-s1.pdf]

Supplementary Infromation

A Novel SRP Recognition Sequence in the Homeostatic Control Region of Heat Shock Transcription Factor σ^{32}

Ryoji Miyazaki[1], Takashi Yura[2], Takehiro Suzuki[3], Naoshi Dohmae[3], Hiroyuki Mori[1], and Yoshinori Akiyama[1†]

[1]Institute for Virus Research, Kyoto University, Kyoto 606-8507, Japan; [2]Faculty of Life Sciences, Kyoto Sangyo University, Kyoto 603-8555, Japan; [3]Center for Sustainable Resource Science, RIKEN, Saitama 351-0198, Japan

[†]Corresponding author: yakiyama@virus.kyoto-u.ac.jp

Supplementary methods

Plasmids

For construction of the pTTQ18-*his*$_6$-*rpoH(amb)* and pTTQ18-*his*$_{10}$-*rpoH(amb)* plasmids, an *amber* mutation was introduced into pRM5 (pTTQ18-*his*$_6$-*rpoH*)[1] and pRM66, respectively, by site-directed mutagenesis. pRM66 (pTTQ18-*his*$_{10}$-*rpoH*) was a derivative of pRM5[1] and was constructed by converting *his*$_6$-*rpoH* to *his*$_{10}$-*rpoH* by site-directed mutagenesis. pTTQ18-*his*$_6$-*rpoH(amb)* derivatives additionally carrying the A50D, K51E, I54N or R91P mutation were constructed by site-directed mutagenesis using an appropriate pTTQ18-*his*$_6$-*rpoH(amb)* plasmid as a template.

pRM26 (pUC118-*ffh*) was constructed as follows. An *ffh* DNA fragment was PCR-amplified from the genome of W3110 using a pair of primers, ffh-for and ffh-rev, digested with EcoRI and BamHI, and cloned into the same sites of pUC118. pRM45 (pTTQ18-*ffh*) was constructed by subcloning the EcoRI-BamHI *ffh* fragment of pRM26 into the same sites of pTTQ18. For construction of pRM52 (pTTQ18-*ffh+ffs*), an *ffs* DNA fragment was PCR-amplified from the genome of W3110 using a pair of primers, ffs-for and ffs-rev, digested with PstI and SphI, and cloned into the same sites of pRM45. pTTQ18-*ffh(amb)+ffs* plasmids were constructed by site-directed mutagenesis using pRM52 as a template. pRM83 was constructed by altering the EcoRI recognition sequence (GAATTC) located outside the multicloning site of pCL1920[2] to TTATTC by site-directed mutagenesis. For construction of pRM83-*ffh+ffs, an ffh+ffs* DNA fragment was PCR-amplified from pRM52 using a pair of primers, srp-for and srp-rev, digested with HindIII and EcoRI, and cloned into the same sites of pRM83. pRM151 (pTTQ18-*ffh(C406S)+ffs*), a plasmid encoding Cys-less Ffh, was constructed by site-directed mutagenesis using pRM52 (pTTQ18-*ffh+ffs*) as a template. The derivatives of pRM151 encoding a single Cys variant of Ffh were constructed by site-directed mutagenesis. For construction of the pSTD689-*ffh(Cys)+ffs* plasmids, the EcoRI-HindIII *ffh(Cys)+ffs*

fragments of the pRM151 derivatives encoding a single Cys Ffh were subcloned into the same sites of pSTD689[3]. pRM203 (pTTQ18-*his$_{10}$-rpoH(T52C)*) was constructed by site-directed mutagenesis using pRM66 (pTTQ18-*his$_{10}$-rpoH*) as a template. pRM153, a derivative of pEVOL-pAzF (Addgene)[4] having a *spc* marker instead of the *cat* marker, was constructed by cloning a *spc* marker fragment that had been PCR-amplified from pHP45Ω[5] using a pair of primers, omega-for and omega-rev, into pEVOL-pAzF using In-Fusion HD Cloning Kit (Takara Bio).

Media and Bacterial Cultures

Cells were grown in L medium (10 g/liter bacto-tryptone, 5 g/liter bacto-yeast extract, 5 g/liter NaCl; pH adjusted to 7.2) or M9 medium (without $CaCl_2$)[6]. 50 µg/ml ampicillin, 20 µg/ml chloramphenicol and 50 µg/ml spectinomycin were added as appropriate for growing plasmid-bearing cells. Bacterial growth was monitored by Mini photo 518R (660 nm; TAITEC) or Klett-Summerson colorimeter (filter No. 54; Klett Manufacturing).

Table S1. Strains used in this study.

Strains	Genotype	References
CAG48238	MG1655 Δ*lacX74* λJW2(P_{htpG}-*lacZ)*	9
CAG48373	CAG48238 Δ*ftsH*::*kan sfhC21 zad-220*::Tn*10*	9
RM591	CAG48238 Δ*dnaKJ*::*kan*	This study
MC4100	*araD139* Δ*(argF-lac)U169 rpsL150 relA1 flbB5301*	10
WAM121	MC4100 ara^{+} *ffh1*::*kan attB*::R6K*ori* P_{araBAD}-ffh^{+} *cat*	11

Table S2. Plasmids used in this study

Plasmids	Vector	Encoded proteins or descritions	References or sources
pTTQ18		Expression vector; P_{tac}, Amp^R	Amersham Pharmacia Biotech
pUC118		Expression vector; P_{lac}, Amp^R	Takara shuzo
pSTD689		Expressopm vector; P_{lac}, Spc^R	3
pCL1920		Expression vector; P_{lac}, Spc^R	2
pRM83		pCL1920 ΔEcoRI outside of MCS	This study
pEVOL-pBpF		p15A-derivative encoding mutant *M. jannaschii* aminoacyl-tRNA synthetase for *p*BPA and the corresponding suppressor tRNA; Cm^R	4
pEVOL-pAzF		p15A-derivative encoding mutant *M. jannaschii* aminoacyl-tRNA synthetase for *p*AzPA and the corresponding suppressor tRNA; Cm^R	4
pRM153		p15A-derivative encoding mutant *M. jannaschii* aminoacyl-tRNA synthetase for *p*AzPA and the corresponding suppressor tRNA; Spc^R	This study
pCP20		pSC101 derivative; *Rep*(Ts) *bla cat* λcI857 λP_R *FLP*$^+$	12
pRM5	pTTQ18	His_6-σ^{32}	1
pRM8	pTTQ18	His_6-σ^{32}(R35*amb*)	This study
pRM9	pTTQ18	His_6-σ^{32}(A38*amb*)	This study
pRM10	pTTQ18	His_6-σ^{32}(L41*amb*)	This study
pRM11	pTTQ18	His_6-σ^{32}(H44*amb*)	This study
pRM12	pTTQ18	His_6-σ^{32}(L47*amb*)	This study
pRM13	pTTQ18	His_6-σ^{32}(E48*amb*)	This study
pRM14	pTTQ18	His_6-σ^{32}(A49*amb*)	This study
pRM15	pTTQ18	His_6-σ^{32}(A50*amb*)	This study
pRM16	pTTQ18	His_6-σ^{32}(K51*amb*)	This study
pRM17	pTTQ18	His_6-σ^{32}(T52*amb*)	1
pRM18	pTTQ18	His_6-σ^{32}(L53*amb*)	This study
pRM19	pTTQ18	His_6-σ^{32}(I54*amb*)	This study
pRM20	pTTQ18	His_6-σ^{32}(L55*amb*)	This study
pRM21	pTTQ18	His_6-σ^{32}(L58*amb*)	This study
pRM22	pTTQ18	His_6-σ^{32}(V61*amb*)	This study
pRM23	pTTQ18	His_6-σ^{32}(I64*amb*)	This study
pRM24	pTTQ18	His_6-σ^{32}(N67*amb*)	This study
pRM26	pUC118	Ffh	This study
pRM28	pTTQ18	His_6-σ^{32}(A50D)	This study
pRM30	pTTQ18	His_6-σ^{32}(K51E)	This study
pRM32	pTTQ18	His_6-σ^{32}(I54N)	This study
pRM34	pTTQ18	His_6-σ^{32}(A50D, K51*amb*)	This study

Table S2. Plasmids used in this study

pRM35	pTTQ18	His_6-σ^{32}(A50D, T52*amb*)	This study
pRM37	pTTQ18	His_6-σ^{32}(K51E, T52*amb*)	This study
pRM39	pTTQ18	His_6-σ^{32}(I54N, K51*amb*)	This study
pRM40	pTTQ18	His_6-σ^{32}(I54N, T52*amb*)	This study
pRM45	pTTQ18	Ffh	This study
pRM51	pTTQ18	4.5S RNA	This study
pRM52	pTTQ18	Ffh and 4.5S RNA	This study
pRM66	pTTQ18	His_{10}-σ^{32}	This study
pRM67	pTTQ18	His_{10}-σ^{32}(K51*amb*)	This study
pRM70	pTTQ18	His_{10}-σ^{32}(L47*amb*)	This study
pRM88	pRM83	Ffh and 4.5S RNA	This study
pRM133	pTTQ18	Ffh(M426*amb*) and 4.5S RNA	This study
pRM135	pTTQ18	Ffh(Q419*amb*) and 4.5S RNA	This study
pRM136	pTTQ18	Ffh(M423*amb*) and 4.5S RNA	This study
pRM139	pTTQ18	Ffh(L319*amb*) and 4.5S RNA	This study
pRM140	pTTQ18	Ffh(K322*amb*) and 4.5S RNA	This study
pRM141	pTTQ18	Ffh(L323*amb*) and 4.5S RNA	This study
pRM142	pTTQ18	Ffh(F334*amb*) and 4.5S RNA	This study
pRM143	pTTQ18	Ffh(L338*amb*) and 4.5S RNA	This study
pRM144	pTTQ18	Ffh(M341*amb*) and 4.5S RNA	This study
pRM145	pTTQ18	Ffh(M344*amb*) and 4.5S RNA	This study
pRM146	pTTQ18	Ffh(M376*amb*) and 4.5S RNA	This study
pRM147	pTTQ18	Ffh(L416*amb*) and 4.5S RNA	This study
pRM148	pTTQ18	Ffh(M427*amb*) and 4.5S RNA	This study
pRM151	pTTQ18	Ffh(C406S) and 4.5S RNA	This study
pRM164	pTTQ18	Ffh(C406S, M341C) and 4.5S RNA	This study
pRM165	pTTQ18	Ffh(C406S, M376C) and 4.5S RNA	This study
pRM166	pTTQ18	Ffh(C406S, M426C) and 4.5S RNA	This study
pRM192	pSTD689	Ffh(C406S) and 4.5S RNA	This study
pRM193	pSTD689	Ffh(C406S, M341C) and 4.5S RNA	This study
pRM194	pSTD689	Ffh(C406S, M376C) and 4.5S RNA	This study
pRM195	pSTD689	Ffh(C406S, M426C) and 4.5S RNA	This study
pRM203	pTTQ18	His_{10}-σ^{32}(T52C)	This study
pRM211	pSTD689	Ffh and 4.5S RNA	This study

Table S3. Primers used in this study

Name	Sequence（5'-3'）
ffh-for	GGGGAATTCGCCAACCGTTTCCACCCCAG
ffh-rev	CCCGGATCCTTAGCGACCAGGGAAGC
ffs-for	GGGCTGCAGTTTTTCCATCTTTTCTTCC
ffs-rev	CCCGCATGCCACGCCGCACAGCCCGTCACG
srp-for	GGCCAAGCTTGCCAACCGTTTCCACCCCAG
srp-rev	GGCCGAATTCCACGCCGCACAGCCCGTCAC
omega-for	GCCACTCATCGCAGTAAAGTGCCACCTGACGTC
omega-rev	TGCTCATCCGGAATTGAGCTGCATGTGTCAGAG

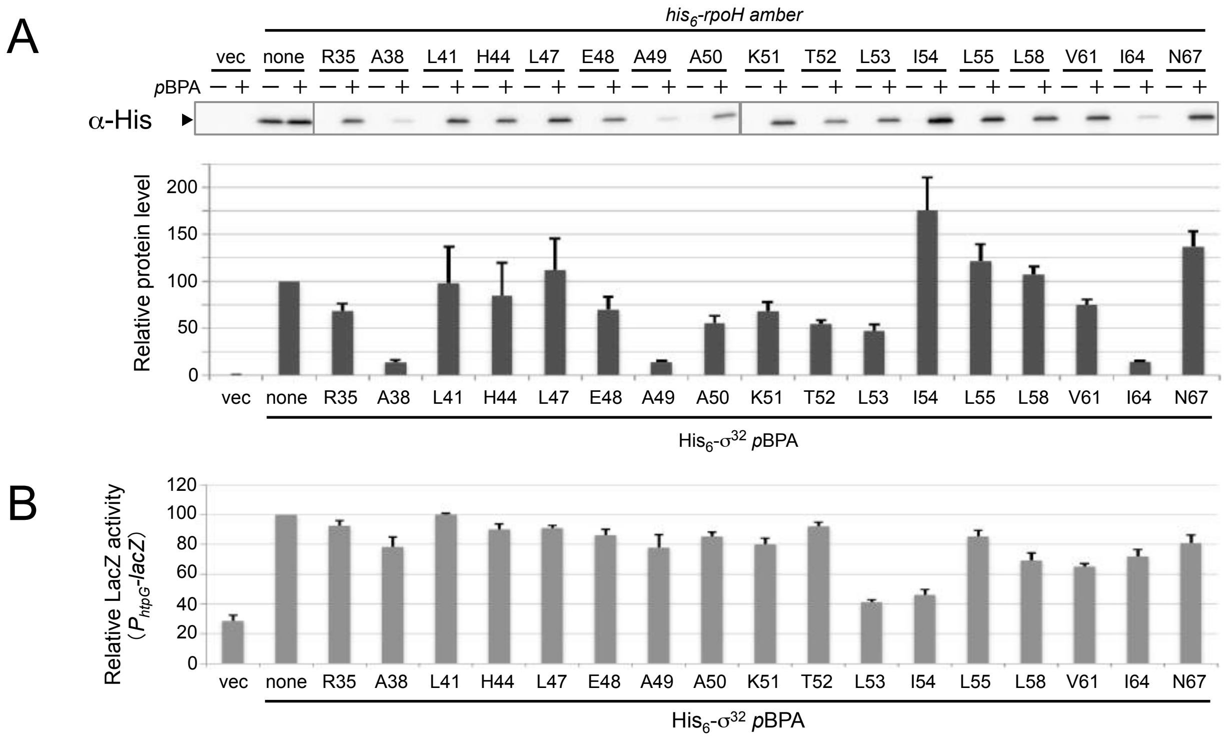

Figure S1. Expression and activities of the His$_6$-σ^{32}*p*BPA variant proteins. (**A**), His$_6$-σ^{32}*p*BPA protein levels. Cells of the strains used in Fig. 2B were grown at 30 ˚C in L-0.02% arabinose medium supplemented with or without 1 mM *p*BPA, and induced with 1 mM IPTG to express His$_6$-σ^{32}*p*BPA for 1 h. Total cellular proteins were analyzed by 10% SDS-PAGE and immunoblotting with anti-His-tag antibodies (*upper panel*). Three independent experiments were performed and mean values of relative band intensities (His$_6$-σ^{32} without *p*BPA was set to 100) are shown along with standard deviations (*lower panel*). (**B**), σ^{32} activity of the His$_6$-σ^{32}*p*BPA proteins. Portion of cells grown with *p*BPA in **A** was taken, and assayed for LacZ activity by the standard procedure[6]. Three independent experiments were performed, and mean values (that for WT was set to 100) are shown along with standard deviations.

A B

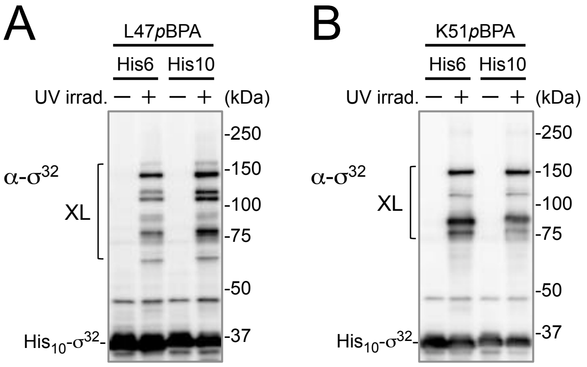

C

XL-1	σ^{32}, DnaK
XL-2	σ^{32}, DnaK, HtpG
XL-3	σ^{32}, HtpG, DnaK
XL-4	σ^{32}, DnaJ, DnaK
XL-5	σ^{32}, DnaK
XL-6	σ^{32}, DnaK, GrpE

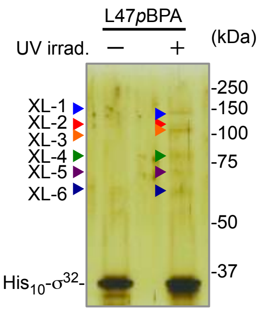

D

XL-1	σ^{32}, DnaK
XL-2	σ^{32}, HtpG, DnaK
XL-3	σ^{32}, DnaK, HtpG, DnaJ, Ffh
XL-4	σ^{32}, DnaJ
XL-5	σ^{32}, SlyD
XL-6	σ^{32}

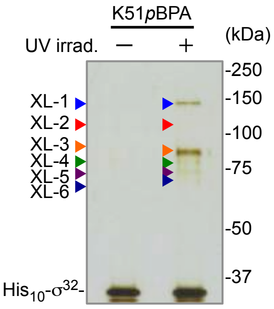

Figure S2. Mass spectrometry (MS) analysis of the σ^{32}*p*BPA cross-linked products. (**A**), (**B**), Cross-linking of the σ^{32}L47*p*BPA (**A**) and σ^{32}K51*p*BPA (**B**) variant proteins carrying His_6- or His_{10}-tag. Cells of CAG48238/ pEVOL-pBpF/pTTQ18-*his_{10}-rpoH (L47amb or K51amb)* were grown at 30°C, induced, and UV-irradiated as in Fig. 2. Total cellular proteins were analyzed by 7.5% SDS-PAGE and immunoblotting with anti-σ^{32} antibodies. (**C**), (**D**), Identification of His_{10}-σ^{32}*p*BPA cross-linking partners by MS analysis. Cross-linked products obtained above with σ^{32}L47*p*BPA and σ^{32}K51*p*BPA (XL-1 to 6, right panel) were affinity purified as described in Methods, separated by 7.5% SDS-PAGE, and subjected to MS analysis. Proteins identified for each XL band are listed on the left. SlyD, rich in His residues, was accidentally purified, independent of His_{10}-σ^{32}.

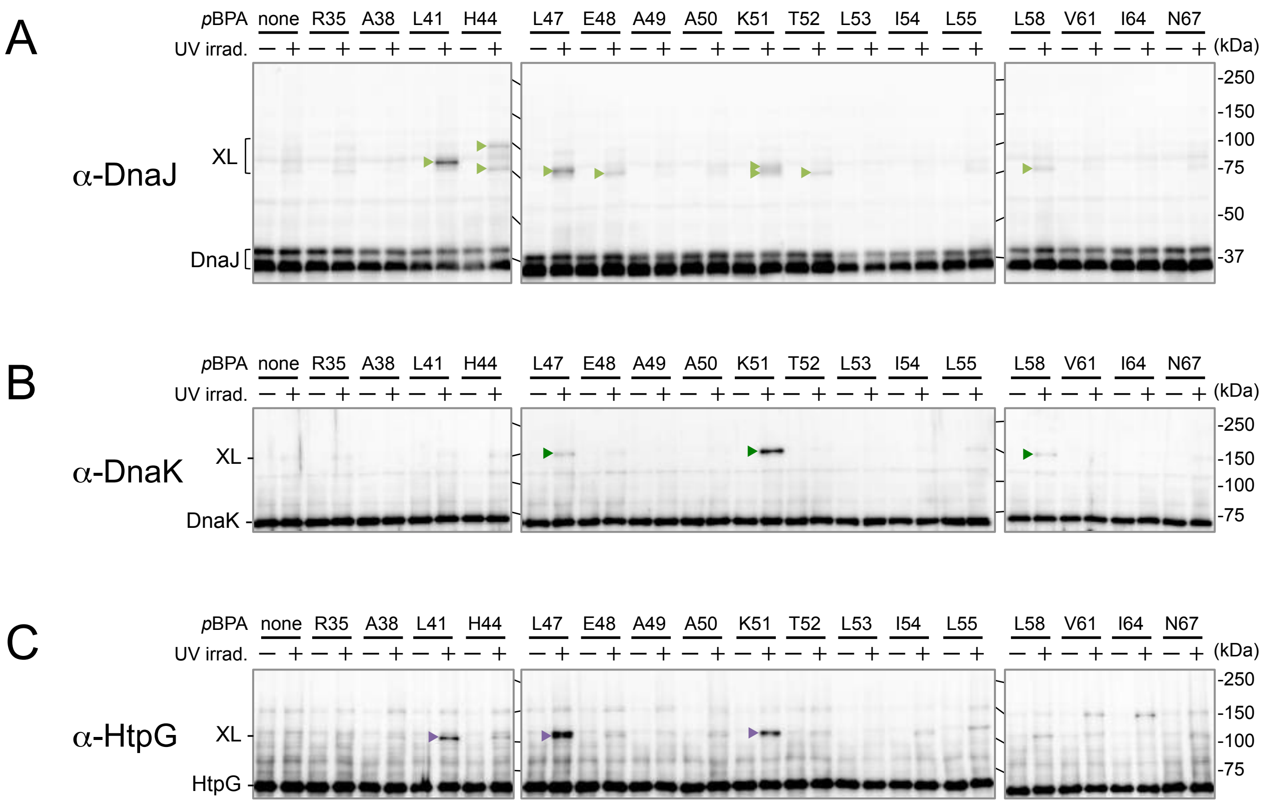

Figure S3. DnaK, DnaJ and HtpG chaperones interacting with the homeostatic control region of σ^{32} were identified by immunoblotting with individual chaperone antibodies. (**A**), (**B**), (**C**), Portions of the samples used in Fig. 2B and C were analyzed by 7.5% SDS-PAGE and immunoblotting with anti-DnaJ (**A**), anti-DnaK (**B**) or anti-HtpG antibodies (**C**). In some positions where these proteins were detected by MS analysis (see Fig. S2), anti-DnaK or anti-HtpG immunoblotting failed to detect the respective protein, probably due to low sensitivities of immunoblotting analysis. Arrow heads indicate cross-linked products.

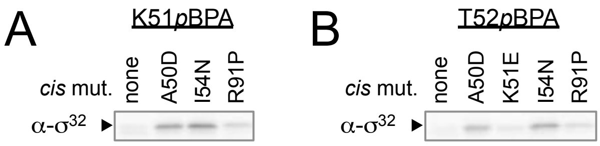

Figure S4. σ^{32} dysregulation mutations enhance protein levels of σ^{32}*p*BPA. (**A**), (**B**), Portion of cellular proteins used in Fig. 3 were analyzed by 10% SDS-PAGE followed by anti-σ^{32} immunoblotting. Typical results using His_6-σ^{32}K51*p*BPA (**A**) and His_6-σ^{32}T52*p*BPA (**B**) with or without a dysregulation mutation are shown.

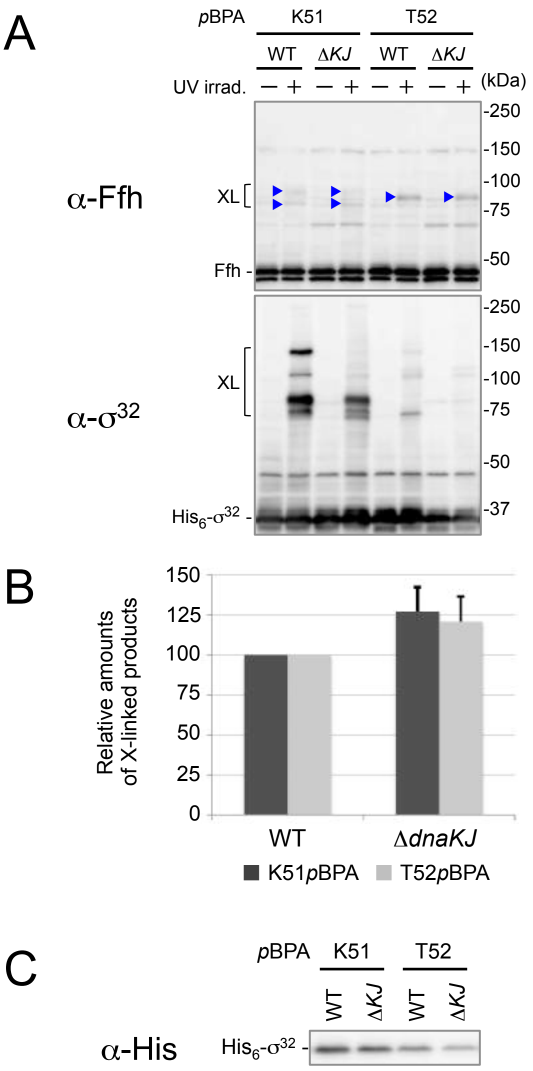

Figure S5. DnaK/DnaJ chaperons are not essential for the σ^{32}–Ffh interaction. (**A**), Cross-linking of σ^{32}*p*BPA in WT and Δ*dnaKJ* strains. Cells of CAG48238 (WT) or RM591 (Δ*dnaKJ*), each carrying pEVOL-pBpF and pTTQ18-*his$_6$-rpoH(amb)*, were grown and analyzed essentially as described in the legend to Fig. 2B and C. (**B**), Quantification of His$_6$-σ^{32}–Ffh cross-linked products. Relative amounts of the cross-linked products detected with anti-σ^{32} antibody in **A** to the amounts of the unirradiated His$_6$-σ^{32} proteins detected with anti-His antibody in **C** were calculated as described in the legend to Fig. 3 (the value for WT was set to 100). Two independent experiments were performed, and mean values are shown along with standard deviations. (**C**), Accumulation of His$_6$-σ^{32} in the Δ*dnaKJ* strain. Portion of cellular proteins in the UV-unirradiated samples used in **A** were analyzed by 10% SDS-PAGE followed by anti-His immunoblotting.

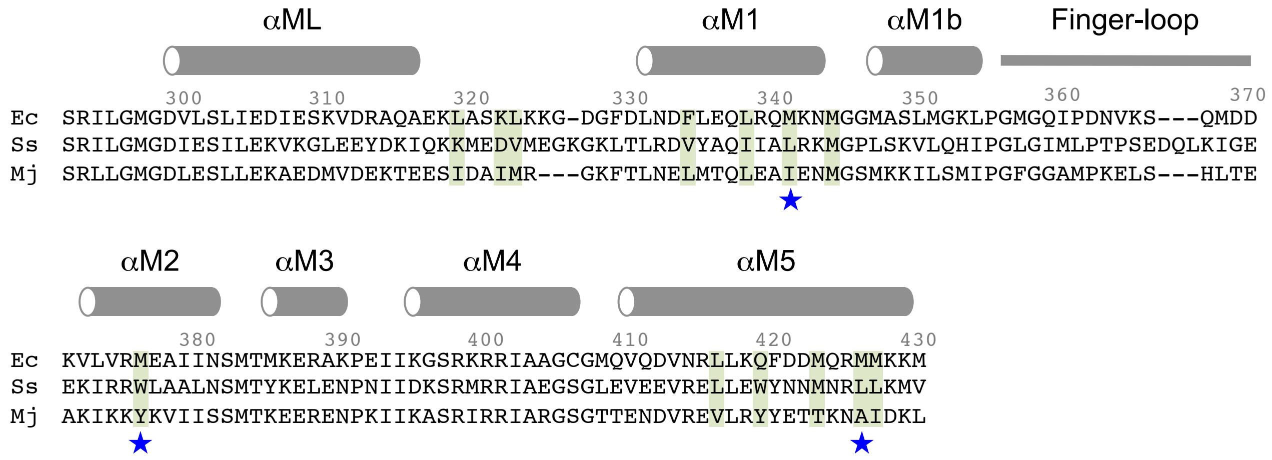

Figure S6. Sequence alignment of *E. coli* Ffh, *S. solfataricus* SRP54 and *M. jannaschii* SRP54. Amino acid sequences of the M domain of *E. coli* Ffh (Ec), *S. solfataricus* SRP54 (Ss) and *M. jannaschii* SRP54 (Mj) are aligned by the ClustalW program. The positions where *p*AzPA was incorporated are colored in light green. Blue stars indicate the positions of cross-linking with σ^{32}. α-helical regions and the finger-loop in the *Ss*SRP54 structure[7] are schematically shown by gray rods and a line, respectively, above the sequences.

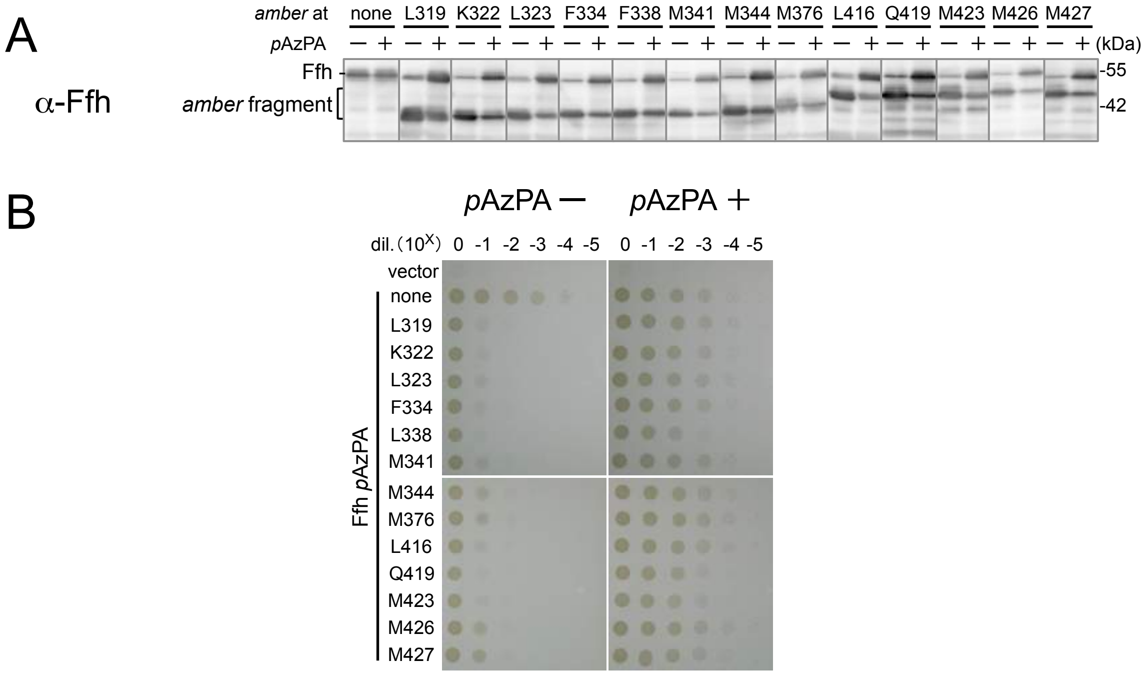

Figure S7. Expression and activities of Ffh*p*AzPA proteins containing *p*AzPA at or around the SP-binding site. (**A**), Protein levels of the Ffh*p*AzPA variants constructed. Samples used in Fig. 5D were analyzed by 10% SDS-PAGE and anti-Ffh immunoblotting. (**B**), Complementation assay for activities of Ffh*p*AzPA proteins. Cells of WAM121 (Δ*ffh1*::*kan* P_{ara}-*ffh*)/pRM153/pTTQ18-*ffh(amb)+ffs* were grown at 30 ˚C in L-medium supplemented with 0.02% arabinose for 3 h, washed and suspended in saline (about 10^9 cells/ml), and serially diluted. Two microliter each of up to 10^5 dilutions was spotted onto L agar plates with or without 1 mM *p*AzPA, and incubated at 30 ˚C for 22 h to observe *p*AzPA-dependent growth of Ffh-depleted cells.

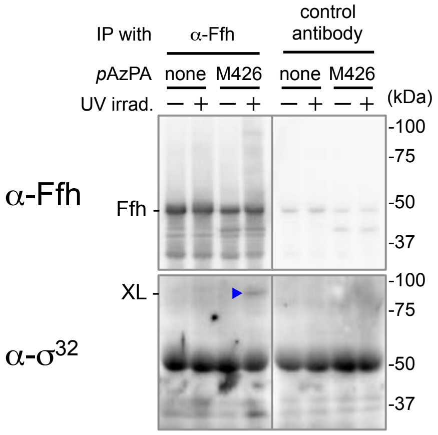

Figure S8. Immunoprecipitation of Ffh*p*AzPA cross-linked products with anti-Ffh and control antibodies. Total cellular protein samples used in Fig. 5E were immunoprecipitated with anti-Ffh or control antibodies, solubilized in SDS sample buffer, and analyzed by 7.5% SDS-PAGE and immunoblotting with anti-σ^{32} and anti-Ffh antibodies.

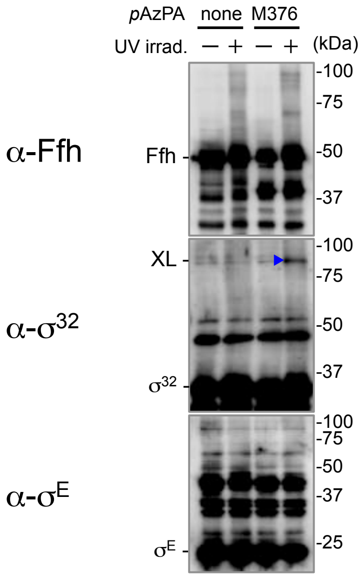

Figure S9. Analysis of cross-linking of Ffh*p*AzPA with σ^E. Immunoblotting analysis of *in vivo* photo cross-linking using Ffh*p*AzPA variant proteins. Cells of CAG48373 (Δ*ftsH sfhC21*)/pEVOL-pAzF/pTTQ18-*ffh(WT or M376amb)+ffs* were grown at 30˚C in L-medium supplemented with 0.02% arabinose and 1 mM *p*AzPA, induced to express Ffh*p*AzPA with 1 mM IPTG for 1 h, and UV-irradiated as indicated. Total cellular proteins were analyzed by 7.5% or 12.5% SDS-PAGE and immunoblotted with anti-Ffh and anti-σ^E antibodies.

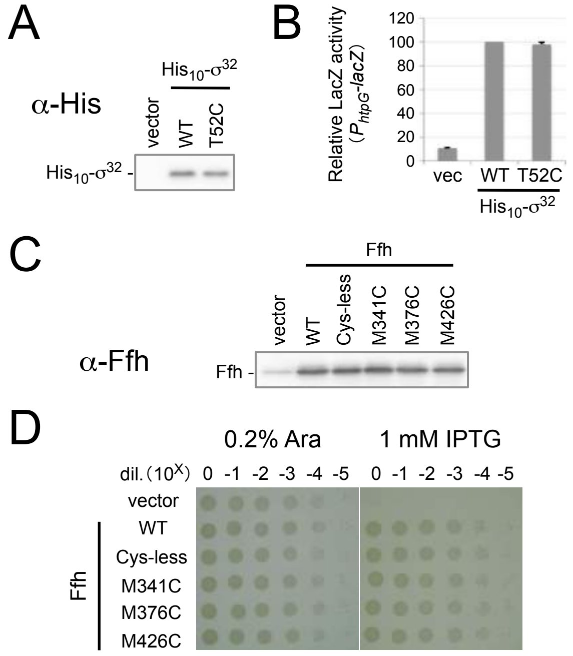

Figure S10. Expression and activities of the single Cys variants of His$_{10}$-σ^{32} and Ffh. (**A**), Protein levels of the His$_{10}$-σ^{32} Cys variant. Cells of CAG48238/pTTQ18-*his$_{10}$-rpoH(Cys)* were grown at 30˚C in L-medium, and induced with 1 mM IPTG to express His$_{10}$-σ^{32} Cys for 1 h. Total cellular proteins were analyzed by 10% SDS-PAGE and immunoblotting with anti-His-tag antibodies. (**B**), σ^{32} activity of the single Cys derivatives of His$_{10}$-σ^{32}. Portion of cells in **A** was taken, and assayed for LacZ activity by the standard procedure[6]. Two independent experiments were performed, and mean values (that for WT was set to 100) are shown along with standard deviations. (**C**), Protein levels of the Ffh Cys variants. Cells of CAG48238/pSTD689-*ffh(Cys)+ffs* were grown at 30˚C in L-medium supplemented with 1 mM IPTG for 2.5 h. Total cellular proteins were analyzed by 10% SDS-PAGE and anti-Ffh immunoblotting. (**D**), Complementation assay for activity of Ffh Cys proteins. Cells of WAM121 (Δ*ffh1*::*kan* P$_{ara}$-*ffh*)/pSTD689-*ffh(Cys)+ffs* were grown at 30˚C in L-medium supplemented with 0.2% arabinose for 3 h. Cells were washed, suspended in saline (about 10^9 cells/ml), and serially diluted. Two microliter each of up to 10^5 dilutions was spotted onto L agar plates supplemented with 0.2% arabinose or 1 mM IPTG, and incubated at 30˚C for 18 h.

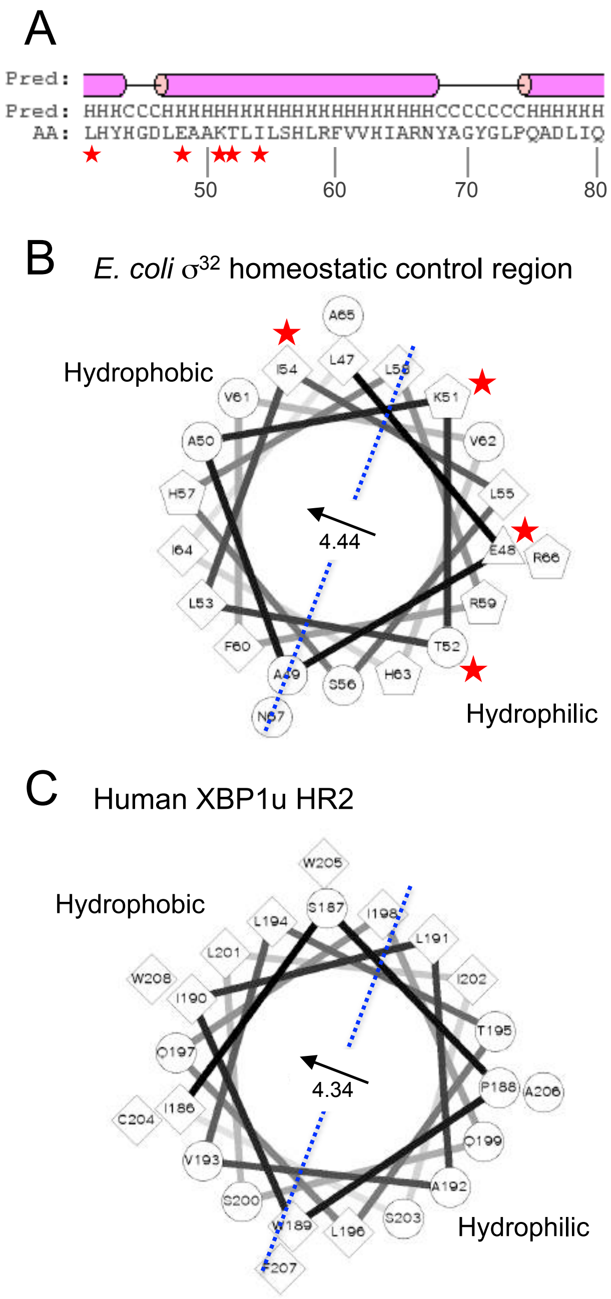

Figure S11. Possible structures of the homeostatic control region of σ^{32} and the HR2 region of XBP1u. (**A**), Secondary structure prediction for σ^{32} region 2.1. The amino acid sequence around the σ^{32} homeostatic control region was used to analyze the secondary structure by the PSIPRED v3.3 program (http://bioinf.cs.ucl.ac.uk/psipred/). Predicted α-helical regions are drawn as pink rods. Red stars indicate the positions of cross-linking with Ffh based on our results (see Fig. 2). (**B**), (**C**), Helical wheel projections of the σ^{32} homeostatic control region (**B**) and the HR2 region of XBP1u (**C**). The sequences of the predicted α-helical regions in the σ^{32} homeostatic control region (residue 47–67) and HR2 (residue 186–208) were subjected to Helical Wheel Prediction analysis (http://rzlab.ucr.edu/scripts/wheel/wheel.cgi). Hydrophilic, hydrophobic, negatively charged, and positively charged residues are shown as circles, diamonds, triangles and pentagons, respectively. The calculated hydrophobic moments[8] for these regions are shown in the center of the projections. Red stars in **B** indicate the positions of cross-linking with Ffh.

SI References

1 Lim, B. *et al.* Heat shock transcription factor σ^{32} co-opts the signal recognition particle to regulate protein homeostasis in *E. coli*. *PLoS Biol.* **11**, e1001735 (2013).

2 Lerner, C. G. & Inouye, M. Low copy number plasmids for regulated low-level expression of cloned genes in *Escherichia coli* with blue/white insert screening capability. *Nucleic Acids Res.* **18**, 4631 (1990).

3 Kanehara, K., Ito, K. & Akiyama, Y. YaeL proteolysis of RseA is controlled by the PDZ domain of YaeL and a Gln-rich region of RseA. *EMBO J.* **22**, 6389-98 (2003).

4 Young, T. S., Ahmad, I., Yin, J. A. & Schultz, P. G. An enhanced system for unnatural amino acid mutagenesis in *E. coli*. *J Mol Biol* **395**, 361-74 (2010).

5 Blondelet-Rouault, M. H., Weiser, J., Lebrihi, A., Branny, P. & Pernodet, J. L. Antibiotic resistance gene cassettes derived from the Ω interposon for use in *E. coli* and *Streptomyces*. *Gene* **190**, 315-7 (1997).

6 Miller, J. H. *Expreiments in Molecular Genetics* (Cold Spring Harbor Lab Press, Cold Sprong Harbor, NY) (1972).

7 Rosendal, K. R., Wild, K., Montoya, G. & Sinning, I. Crystal structure of the complete core of archaeal signal recognition particle and implications for interdomain communication. *Proc. Natl. Acad. Sci. USA* **100**, 14701-6 (2003).

8 Eisenberg, D., Weiss, R. M. & Terwilliger, T. C. The hydrophobic moment detects periodicity in protein hydrophobicity. *Proc. Natl. Acad. Sci. USA* **81**, 140-4 (1984).

9 Yura, T. *et al.* Analysis of σ^{32} mutants defective in chaperone-mediated feedback control reveals unexpected complexity of the heat shock response. *Proc. Natl. Acad. Sci. USA* **104**, 17638-43 (2007).

10 Silhavy, T. J., Berman, M. L. & Enquist, L. W. Experiments with Gene Fusions (Cold Spring Harbor Lab Press, Cold Spring Harbor, NY). (1984).

11 de Gier, J. W. *et al.* Assembly of a cytoplasmic membrane protein in *Escherichia coli* is dependent on the signal recognition particle. *FEBS Lett.* **399**, 307-9 (1996).

12 Cherepanov, P. P. & Wackernagel, W. Gene disruption in *Escherichia coli*: Tc^R and Km^R cassettes with the option of Flp-catalyzed excision of the antibiotic-resistance determinant. *Gene* **158**, 9-14 (1995).
